# Effectiveness of Positive Psychotherapy on Depression and Alexithymia in Women Applying for a Divorce

**DOI:** 10.1155/2022/8446611

**Published:** 2022-02-16

**Authors:** Diana Khalili, Nadia Khalili, Eisa Jafari

**Affiliations:** ^1^Payame Noor University, Iran; ^2^Department of Educational and Counselling Psychology, McGill University, Montreal, Canada

## Abstract

**Background:**

The new therapeutic approach of positive psychotherapy has successfully treated severe mental disorders such as depression and mood disorders. However, existing research has not sufficiently measured the usefulness of this treatment in reducing depression and alexithymia.

**Objectives:**

This study thus examined the effectiveness of positive psychotherapy in reducing these two conditions in a specific population: Iranian women applying for the divorce.

**Methods:**

A total of 40 participants aged 20-40 with a high score in the Beck Depression Inventory and Toronto Alexithymia Questionnaire were recruited from women referred to a psychology clinic for divorce-related problems. The pretest, posttest, and follow-up were conducted with all participants, who were randomly placed in two groups: the experimental and control groups, which each consisted of 20 people. We provided eight positive psychotherapy sessions for only the experimental group.

**Results:**

After MANCOVA was conducted, the results showed that positive psychotherapy significantly decreased alexithymia and depression in the test population.

## 1. Introduction

The family is considered the smallest and most crucial unit of society, which could positively and negatively influence its members [1]. Previous studies have suggested that divorce is one of the most harmful effects on a family; divorce can heighten mental health risks for individuals and the family by significantly decreasing their well-being [2–4]. This multidimensional phenomenon can cause and intensify physical and psychological disorders such as neurological, cognitive, emotional, and behavioral illness [5].

Divorce/separation, as one of the most common results of dissatisfaction among couples, develops an emotional disruption for one or both members of the couple [2, 6]. This emotional breakdown is undoubtedly frustrating and is accompanied by different traumas for couples. In other words, divorce is a process that begins with both members of the couple experiencing the emotional crisis and ends with them trying to resolve the conflict by entering a new position with new roles and lifestyles [7]. Recently, the rate of global divorce has swiftly increased due to rapid social, economic, and cultural transformation [8–11], even in religious countries with substantial social and legal obstacles for divorce such as Iran [12, 13]. Sadeghi and Agadjanian [13] suggest the following:

Although divorce is permitted in Islam, Islamic law discourages Muslims from seeking a divorce, especially for minor reasons. In the Islamic Republic of Iran, marriage and family remain very strong social institutions. Formation and maintenance of marriage are culturally and socially encouraged, and divorce has long been condemned as incompatible with family and societal values. However, in the context of structural and ideational transformation of Iranian society, the stigmatization of divorce has declined, and divorce has become both legally and socially easier. As a result, divorce rates in Iran have increased rapidly since the mid-1990s. Although divorce rates are still low in Iran compared to Western societies, the increasing trend in divorce and its high concentration at young ages deserve attention (p. 479, 480).

In this context, divorced people, specifically women, experience high social and cultural pressure that can endanger their mental health, leading to psychological problems such as depression, anxiety, and emotional disturbances [14].

Researchers reported depression as one of the most prevalent disorders in divorced/separated women and men who have lost intimate and emotional relationships [15]. Still, studies have shown that women with stress experience more negative emotions than men, and the prevalence of depression is higher in women than men [16]. Asadpour and Hosseini [17] also illustrated that divorced women often suffer from negative feelings such as guilt, depression, and distress; according to Chen et al. [18], those who lose their intimate relationships, in addition to experiencing depression, would face more severe problems such as drug and alcohol abuse and suicidal thoughts.

In the latest edition of the Diagnostic and Statistical Manual of Mental Disorders (DSM-5), diagnostic criteria for depression include depressed mood, lack of pleasure or a sharp drop in interest, a 5% decrease or increase in weight, sleep disturbances such as sleeplessness or oversleeping, restlessness or mental-physical dullness, exhaustion, feelings of guilt and worthlessness/incompetence, loss of concentration, doubts, and recurrent thoughts about death or suicide. These symptoms affect the social-occupational functions and other vital aspects of patients [19], including the intimate relationships of couples. The condition of alexithymia, which is described as a set of cognitive and emotional features observed among patients with mental disorders [20], is, therefore, an influential variable affecting couples and contributing to divorce. Alexithymia has been studied concerning the processing and adjustment of emotions [21], and alexithymia characteristics have been shown to pave the way for interpersonal and marital life problems [22]. As emotion regulation can positively influence alexithymia, it can consequently affect coupled life and divorce as well, in ways that have so far been overlooked [23].

Multidimensional structural alexithymia consists of four distinct characteristics: (a) having trouble recognizing and describing emotions, (b) the difficulty in distinguishing between emotions and physical stimuli, (c) deficient fantasies, and (d) objective and inferior introverted thinking (externally oriented thinking) [24]. The definition of alexithymia features contrasts with effective emotional regulation, and studies have demonstrated the relationship between alexithymia and inefficient emotional regulation [25]. To illustrate this point, people with alexithymia symptoms are more likely to use suppression strategies than asymptomatic people and less likely to use reappraisal strategies. Of the two approaches mentioned, suppression strategies are more closely related to physical and mental health problems and thus considered maladaptive to regulate emotion [18]. The efforts of psychologists to understand mood disorders and how they are formed and treated have led to the development of various treatment theories and methods around biological and psychosocial areas. Over the past several years, therapeutic approaches often had a pathologic view of disorders and aimed at relieving and appeasing disease symptoms. Yet, in the past two decades, new approaches such as positive-oriented psychology have been widely used. Lee et al. [26] argue that psychology considers only morbid and pathologic aspects. Not only have psychologists paid insufficient attention to the abilities, positive aspects, and inner virtues of human beings, but they have also solely considered harms [27]. Peterson [28] believes that focusing on disorders enabled psychologists to identify ways to resolve them, but with a short-sighted understanding of human nature. The positive psychology approach, on the other hand, fully considers the positive facets of life, including the psychological assets and strengths of individuals [29]. Senf and Liau [30] argue that strength-based interventions have been shown to increase happiness and reduce depression. Likewise, Pietrowsky and Mikutta [31] also state that positive psychology effectively reduces depression.

Previous studies mainly have focused on separated/divorced people and the psychological effect of various therapists' methods to cope with such problems. Yet, less is known about the psychological problems of people who apply for divorce and the effect of therapy on them. Given this research gap and the higher pressure that the divorce process places on Iranian women compared to men [32], the following study will determine how positive psychotherapy affects depression and alexithymia in women applying for a divorce. More specifically, this study is aimed at knowing if a positive psychotropic workshop consisting of eight sessions can decrease depression and alexithymia among Iranian women who have applied for a divorce. We hypothesize that (1) alexithymia and its subscales as well as depression significantly decrease among this population after the therapy sessions. (2) All three subscales of alexithymia, having trouble recognizing emotion, difficulty in describing emotion, and externally oriented thinking, are positively influenced by therapy sessions.

## 2. Method

The present study uses quantitative data collection methods, with a semiexperimental design including pretest and posttest questionnaires for both experimental and control groups. After receiving ethic board confirmation, recruitment started.

### 2.1. Participants

The sample of the study was a group of women (aged 20 to 40) applying for a divorce, who was referred to a psychology clinic in Karaj City, Iran in 2019. The total number of these women was 230 at first, which meets Morgan's table, and the sample size for the initial screening was 140 women. After the required coordination with the psychology clinic, 40 women, who had the highest scores on the Beck Depression Inventory (BDI) and the Toronto Alexithymia Scale (TAS 20), were willingly selected. Out of an original sample of 230 women, an initial screening of 140 women was conducted by Morgan's table.

### 2.2. Procedure

All participants (*N* = 40) were randomly divided into two groups, the first one (*N* = 20) as the control group and the second (*N* = 20) as the experimental group. Depression and alexithymia pretests were taken from both groups in coordination with the psychological clinic. Subsequently, positive psychotherapy was offered to the experimental group in eight sessions (90 minutes per session) and about two months (details of the sessions are summarized in Table 1.) The control group underwent no intervention or special treatment during this period. After completing the training for the experimental group, Beck Depression Inventory and Toronto Alexithymia Scale posttests were implemented for both groups. Finally, research hypotheses were appraised and concluded based on pretests and posttests.

During the distribution of questionnaires, it was ethically imperative to assure respondents that all the completed information would be kept confidential by the researchers and be included anonymously in the article. Additionally, after completing the research, people in the control group received an educational package. Thus, the researchers gave the participants a summary of training sessions and bestowed gifts in appreciation of their attendance. After data collection, a multivariate analysis of variation (MANCOVA) with the SPSS 25 software was used to interpret the collected data. We designed the content of the training sessions after consulting with professors and specialists in the field of positive psychology and by sources such as *Positive Psychology Techniques* [33], *Positive psychology progress: empirical validation of interventions* [34] *Inner Happiness* [35], *Learned Optimism* [36], and *Positive Psychotherapy* [37] (see Table 1).

### 2.3. Ethical Considerations

1. The ethical principles of clinical and general psychology were considered in this article. Participants were aware of the research process and allowed to leave the sessions whenever they wished
2. The researcher was committed to preserving the anonymity of the participants
3. Research results were provided to participants upon request
4. Participants in the control and experimental groups did not participate in any course of treatment and psychological intervention during the intervention simultaneously
5. A positive psychotherapy workshop was provided to control group members after the completion of the research process

### 2.4. Beck Depression Inventory

Beck's Depression Inventory (BDI) [38] has been validated as a screening test for the severity of depression symptoms [39]. The test involves 21 self-report questions assessing the participant's symptoms in the past two weeks. As an effective tool in clinical and nonclinical specimens, BDI possesses a high internal consistency, test-retest reliability, and both differential and synchronized validation ns [40]. BDI's reliability is 0.93, and its validity is 0.77, while its internal consistency is *α* = 0.91. Its minimum and maximum final scores with summing up points are zero and 63, respectively. Based on the scores, the individual's status is assessed as 5-9: normal range, 10-18: mild to moderate depression, 19-29: mild to severe depression, 30-63: severe depression, and below 4: pretending to be patient, histrionic/interstitial personality, while expressing depression [41].

### 2.5. Alexithymia Scale

The Toronto Alexithymia Scale (TAS 20), is a 5-Likert scale questionnaire developed by [42] that evaluates alexithymia and its three subscales through 20 questions: (1) difficulty identifying feelings (DIF: 7 questions), (2) difficulty describing feelings (DDF: 5 questions), and (3) external-oriented thinking (EOT: 8 questions). Assessing the scale's applicability for the Iranian community, [43] reported the Iranian sample's validity as 0.71 using the half-split method and the reliability as 0.83 using a retest. The original test's validity and reliability were then determined to be 0.85 and 0.84, respectively.

## 3. Results

### 3.1. Descriptive Findings

Descriptive results (listed in Table 2) demonstrated that the mean of depression in the control group (*N* = 20) was 47.05 and 45.9 for the pretest and posttest, respectively, while the posttest means, 32.95, was less than the pretest means of 45.45 in the experimental group (*N* = 20).

### 3.2. Assumption Check

Multivariate analysis of variance (MANCOVA) was conducted to determine the effectiveness of independent variables for linear composition. Moreover, in this method to control the effect of pretests as a random variable, the effect of the pretests was controlled by sphericity. All assumptions were checked and met the criteria.

The correlation coefficient between depression scores, alexithymia, and its subscales in the posttest with the Pearson correlation coefficient was examined. The findings in Table 3 showed that there is a significant correlation between depression and alexithymia and its subscales in women. Hence, the assumption of a moderate correlation between dependent variables is established.

To check the homogeneity of the variance matrix and covariance, Box's M test was used to check the homogeneity. Considering that the significance level is 0.30 > 0.05, the null hypothesis is not rejected, *F* (3.259920) = 1.22, *P* > 0.05. Thus, the homogeneity of the variance matrix and covariance of the variables is met.

Leven test was used to test the equality of variances. And because depression (*P* < 0.05, *F* = 1.41 (2.38)) and emotional malaise (*P* < 0.05, *F* = 1.75 (2.38)) were significantly greater than the significance level of *α* = 0.05, at this level, the null hypothesis is not rejected and as a result, it can be concluded that the variance between the control and experimental groups in both dependent variables is equal.

Kolmogorov-Smirnov (K-S) test was used to test the normality of the distribution of scores. And because the significance of depression (*P* = 0.20) and emotional malaise (*P* = 0.21) is greater than the significance level of *α* = 0.05, so, at this level, the null hypothesis is not rejected and as a result, the distribution of scores in both variables has a normal distribution. Given that all four assumptions of multivariate analysis of variance (MANOVA) are established, thus, we are allowed to use multivariate analysis of variance to test the research hypothesis.

### 3.3. Multivariate Analysis of Variance (MANCOVA)

The results of multivariate analysis of variance (MANOVA) in Table 4 show that the null hypothesis is rejected for all hypotheses (*F*_(2, 35)_ = 255.12, *P* < 0.05, lambda = 0.064, and ȵ^2^ = 0.936). Therefore, positive psychotherapy has a significant effect on alexithymia and its three subscales, as well as depression, in women applying for a divorce.

The results obtained in Table 5 show that the control and experimental groups in the posttest of depression (*F*_(1.39)_ = 215.50, *P* < 0.05, and ȵ^2^ = 0.857) and alexithymia (*F*_(1.39)_ = 170.78, *P* < 0.05, and ȵ^2^ = 0.826) were significantly different. Positive psychotherapy simultaneously had a significant effect on the linear combination of depression and alexithymia in women applying for a divorce. The effect of positive psychotherapy on depression (0.857) and alexithymia (0.826) was notable. Likewise, the control and experimental groups in the posttest of the three alexithymia subscales were significantly different. Positive psychotherapy had a greater effect on external-oriented thinking (*F*_(1.39)_ = 215.50, *P* < 0.05, *ȵ*2 = 0.857) than on difficulty identifying feelings (*F*_(1.39)_ = 215.50, *P* < 0.05, and ȵ^2^ = 0.857) and difficulty describing feelings (*F*_(1.39)_ = 215.50, *P* < 0.05, and ȵ^2^ = 0.857). These results indicate that positive psychotherapy simultaneously had a significant effect on the linear combination of (a) difficulty identifying feelings (DIF), (b) difficulty describing feelings (DDF), and (c) external-oriented thinking (EOT).

### 3.4. Multivariate Analysis of Variance (MANCOVA)

The results of multivariate analysis of variance (MANCOVA) in Table 6 show that the null hypothesis is rejected ( = 2 = 0.834, lambda = 0.166, *P* < 0.05, and *F* = 22.22 (2, 33)). Therefore, positive psychotherapy can be said to have a significant effect on the difficulty in identifying and describing emotions and objective thinking.

The results obtained in Table 7 show significant differences in control and experimental groups for the three alexithymia subscales in the posttest. Participants in the experimental group showed a significant difficulty in identifying emotions, difficulty in describing emotions ( = 2 = 0.391, *P* < 0.05, and *F* = 22.50 (1.39)). And for objective thinking ( = 2 = 0.483, *P* < 0.05, and *F* = 32.73 (1.39)) in other words, positive psychotherapy had a considerable effect on the linear combination of the participants' difficulty identifying and describing emotions with their objective thinking. The effect of positive psychotherapy on the difficulty in identifying emotions was 0.17, while the effect on difficulty in describing emotions was 0.391, and the effect on objective thinking was 0.483.

## 4. Discussion and Conclusion

The results support our first hypothesis that positive psychotherapy alleviates depression in women aged 20 to 40 who are applying for a divorce. These findings are in line with a meta-analysis study by Bolier et al. [44], as well as research by Lyubomirsky and Layous [45], Jabbari et al. [46], and Guo et al. [47], which showed that positive psychology interventions relieved depression symptoms in the adult population, including women. Our results also support our second hypothesis that positive psychotherapy interventions reduce alexithymia in women applying for a divorce in Iran, which is similar to the findings of Nejat et al. [48] and Sanagouye Moharer et al. [49], who suggested that positive psychotherapy reduces alexithymia in Iranian women who were referred to psychologists for couple therapy, as well as female adolescents in Iranian schools. Our results specifically showed that positive psychotherapy decreases the alexithymia subscales of difficulty identifying feelings, difficulty describing feelings, and external-oriented thinking. Our results were further consistent with previous findings that positive psychology interventions can be effective in the enhancement of subjective well-being and psychological well-being, as well as in helping to reduce depressive symptoms [29–31].

Descriptive results showed a remarkable decline in posttest depression scores of the experimental group, which were significantly higher than in previous studies. These findings illustrate that depression decreases as a result of positive psychotherapy when individuals accept reality, view themselves as unbiased, practice self-compassion, and overcome daily life difficulties and stresses. These results support past studies that optimism is correlated positively with coping strategies in managing and decreasing stress and abandonment [50]. The present study further shows that positive psychology does not exclusively focus on positivist aspects of human experience. Positive psychotherapy considers all sorts of emotional experiences that cause depression, such as feelings of unpleasantness. This method also approaches pain empathetically, gently encouraging people to seek meaning and psychological growth in unfortunate events, while providing coping skills. To clarify, current findings suggest that through positive psychology, women applying for divorce are heartened to recognize their positive aspects and able to express their emotions. They evolve their abilities, competencies, and coping skills to improve their health. Because of the study's positive findings, we suggest performing positive psychotherapy in the counseling, treatment, and practical aspects of psychology.

### 4.1. Limitation

We acknowledge that our study had several limitations, including small sample size, not providing any treatment to the control group during the study, and the lack of a follow-up test to measure the effect of positive psychology after several months. Furthermore, participants of the study were recruited from one of the Iranian cities, which was not representative of cultural differences within Iran. Conducting similar research on men applying for divorce could be also beneficial for comparison. Finally, we suggest future investigations of positive psychotherapy's effectiveness on remaining essential variables such as marital life quality, resilience, mindfulness, and sexual intimacy of women applying for divorce.

## Figures and Tables

**Table 1:** Summary of positive psychotherapy.

Number	Topic	Objectives and summary of meetings
Session 1	Introduction—the concept of positivity-initial evaluation	We begin with a general discussion about the meaning and concept of positive thinking, explaining the purpose of the sessions and how they will run. Participants receive a brief explanation of positivity, coping styles, and related theories.

Session 2	Happiness skills and social skills	After reviewing the previous session's assignments on strength identification, we discuss the concept of happiness and techniques to enhance its (i) Healthy diet and exercise (ii) Changing attitudes and lifestyles (iii) Developing altruistic behavior and social relations (iv) Keeping a journal of positive memories (e.g., writing about three good things that happen every day) We end the session by discussing social relationships, obstacles to their development, and techniques for enhancing them.

Session 3	The forgiveness process model	We first review the previous session's assignments on the concept of happiness and then discuss forgiveness, and then, the participants write a sample letter of forgiveness. Instructors give the participants advice on how to improve their letters.

Session 4	Gratitude-optimism	We review the previous topic and introduce the concept of gratitude, its benefits, and methods of expressing it. Participants write a gratitude letter and then discuss the concept and benefits of optimism. Instructors demonstrate how to improve the letters and then present the ABCDE assignments. Next, we discuss the source of control.

Session 5	Hope and purposefulness	After a review of optimism, we explore the concept of hope and its importance. Instructors help participants to specify and set their goals and how to achieve them, identify obstacles in their path, and overcome them.

Session 6	Self-esteem, self-worth, and self-efficacy	We review the previous topic and then explain the benefits of self-esteem, self-worth, and self-efficacy. Instructors explain how to develop these feelings and positive coping strategies (e.g., problem-solving, social support, and acquiring new skills) by paying attention to participants' strengths).

Session 7	Living a meaningful life	After reviewing the previous topic, instructors explain the benefits of a meaningful life and the importance of goals to achieve it. Participants practice how to find meaning in their lives through laughter, self-confidence, and healthy fitness habits.

Session 8	Closing session	After briefly summarizing all of the previous sessions and thanking participants for their contributions, instructors run posttests.

**Table 2:** Descriptive statistics of research variables in control and experimental groups in pretest and posttest.

Variable	Group	Pretest		Posttest	
		Mean	SD	Mean	SD
Depression	Experiment	45.45	5.14	32.95	4.24
	Control	47.05	5.21	45.9	5.14

Alexithymia	Experiment	58.80	7.10	48.85	4.95
	Control	59.55	6.72	58.65	6.87

DIF	Experiment	20.95	3.27	19.1	2.27
	Control	22.15	3.01	21.1	3.06

DDF	Experiment	14.15	3.01	12.15	2.11
	Control	14.85	3.36	15.1	2.15

EOT	Experiment	23.70	3.71	17.6	4.21
	Control	22.55	5.11	22.45	4.26

**Table 3:** Pearson correlation test statistics are related to the relationship between depression and alexithymia.

Variable	Depression	Alexithymia
Depression	1	-
Alexithymia	0.515	1

**Table 4:** Multivariate tests.

Effect		Value	*F*	Hypothesis df	Error df	Sig.	Partial eta squared
Depression	Pillai's trace	.761	55.77	2	35	.000	.761
	Wilks' lambda	.239	55.77	2	35	.000	.761

Alexithymia	Pillai's trace	.889	139.93	2	35	.000	.889
	Wilks' lambda	.111	139.93	2	35	.000	.889

Group	Pillai's trace	.936	255.12	2	35	.000	.936
	Wilks' lambda	.064	255.12	2	35	.000	.936

**Table 5:** Tests of between-subject effects.

Source	Dependent variable	Type III sum of squares	df	Mean square	*F*	Sig.	Partial eta squared
Group	Depression	1300.79	1	1300.79	25.50	.000	.857
	Alexithymia	802.51	1	802.51	170.78	.000	.826

Error	Depression	217.30	36	6.04			
	Alexithymia	169.16	36	4.70			

Total	Depression	64685	40				
	Alexithymia	117888	40				

Corrected Total	Depression	2511.77	39				
	Alexithymia	2325.50	39				

**Table 6:** Multivariate tests.

Effect		Value	*F*	Hypothesis df	Error df	Sig.	Partial eta squared
DIF.pre	Pillai's trace	.579	15.135	3	33	.000	.579
	Wilks' lambda	.421	15.135	3	33	.000	.579
	Hotelling's trace	1.376	15.135	3	33	.000	.579
	Roy's largest root	1.376	15.135	3	33	.000	.579

DDF.pre	Pillai's trace	.626	18.420	3	33	.000	.626
	Wilks' lambda	.374	18.420	3	33	.000	.626
	Hotelling's trace	1.675	18.420	3	33	.000	.626
	Roy's largest root	1.675	18.420	3	33	.000	.626

EOT.pre	Pillai's trace	.768	36.349	3	33	.000	.768
	Wilks' lambda	.232	36.349	3	33	.000	.768
	Hotelling's trace	3.304	36.349	3	33	.000	.768
	Roy's largest root	3.304	36.349	3	33	.000	.768

Group	Pillai's trace	.834	55.216	3	33	.000	.834
	Wilks' lambda	.166	55.216	3	33	.000	.834
	Hotelling's trace	5.020	55.216	3	33	.000	.834
	Roy's largest root	5.020	55.216	3	33	.000	.834

**Table 7:** Tests of between-subject effects.

Source	Dependent variable	Type III sum of squares	df	Mean square	*F*	Sig.	Partial eta squared
Group	DIF.pre	45.205	1	45.205	7.161	.011	.170
	DDF.pre	63.236	1	63.236	22.496	.000	.391
	EOT.pre	189.241	1	189.24	32.727	.000	.483

Error	DIF.post	220.948	35	6.313			
	DDF.post	98.386	35	2.811			
	EOT.post	202.387	35	5.782			

Total	DIF.post	16476.000	40				
	DDF.post	7685.000	40				
	EOT.post	16957.000	40				

## Data Availability

The data used to support the findings of this study are available from the corresponding author upon request.
